# Erythrodermic atopic dermatitis with late onset–case presentation

**Published:** 2010-02-25

**Authors:** C Lancrajan, R Bumbacea, C Giurcaneanu

**Affiliations:** * Department of Oncologic Dermatology and Allergology, Elias University Emergency Hospital, BucharestRomania; ** ‘Carol Davila’ University of Medicine and Pharmacy– BucharestRomania

## Abstract

Atopic dermatitis is a chronic inflammatory disease, usually associated with a personal or family history of atopic diseases such as AD, allergic rhinitis or asthma that most commonly arise in childhood and present with elevated IgE serum in up to 85% of patients. The severity of AD is based on the extent of affected areas, itch intensity and appearance of skin lesions.

Here, we present the case of a 21–year–old female patient with generalized erythematous eczematous skin lesions, flexural lichenifications accompanied by intense pruritus, painful fissures and erosions resulting from scratching. She also presented erythematous plaques with thin scales on the scalp. The patient had no personal or familial history of AD, allergic rhinitis or asthma and the onset of cutaneous symptoms presented severe exacerbation in the last 2 months of the last 3 years. The main laboratory findings were–high serum eosinofilia (2,400/µL) and very high total IgE serum (11449UI/L). The flare remission was induced with systemic treatment (corticotherapy, oral H1 antihistamines, and antibiotherapy) and topical therapy (UVB 311nm, topical glucocorticoids and hydration).

It is very important to recognize AD as a cause of erythroderma, especially in a patient with a late onset of the disease, in order to treat it promptly and to prevent ulterior recurrences, by educating the patient to have an adequate life style and to treat the recurrences at the very first symptoms.

## Introduction

Atopic dermatitis is an inflammatory, relapsing, chronic skin disease that usually begins in infancy and it presents with dry skin, erythematous–edematous and veziculous papules and plaques accompanied by intense pruritus. Rubbing and scratching lead to erosions, fissures and lichenification. The predilected sites are flexures, neck, face, eyelids, wrists and dorsa of feet and hands or can be generalized in severe disease [1]. The lesions are easily colonized with *Staphylococcus aureus* which exacerbates the skin inflammation and maintains the vicious cycle by secreting exotoxins that may act as superantigens stimulating the activation of T cells and macrophages [1,2].

Atopic dermatitis is frequently associated with a personal or family history of atopic diseases such as atopic dermatitis, allergic rhinitis or asthma. Significant proportions of affected children have persistent atopic dermatitis after puberty and are at risk of developing respiratory allergies. The underlying pathophysiologic and genetic mechanisms are yet unknown but involve interactions between genetic factors, the immune system and the environment [3].

The diagnosis is based on clinical aspects of the lesions, the most frequently used criteria being those proposed by Hanifin and Rajka. Three of four major criteria are necessary–pruritus, typical morphology and distribution, chronically relapsing course and atopic personal or family history, in addition to three other signs of atopy like xerosis, keratosis pilaris, palmar hyperliniarity, Dennie–Morgan infraorbital fold, periocular pigmentation, the Hertoghe sign of the lateral eyebrow, white dermografism, cheilitis, conjunctivitis, keratoconus, subcapsular anterior cataract [1].

Food allergy is an important trigger of atopic dermatitis. Skin prick tests or specific IgE serum have a high negative predictive value when they are negative. When they are positive they should be followed by placebo controlled oral food challenges in patients without history of life threatening reactions after the ingestion of specific food for preventing unnecessary dietary limitations [4–9]. Skin prick tests or specific IgE serum are useful to determine sensitivity to inhalant allergens such as house dust mite and animal dander [5,10– 12]. Up to 85% of patients have an elevated IgE serum level.

The grading of the severity of atopic dermatitis is based on the extent of affected areas, itch intensity and appearance of skin lesions. The disease can complicate with erythroderma and risk of exfoliation, ocular complications such as keratoconus or keratoconjuctivitis sleep disturbances, psychological disturbances, and bacterial or viral skin infections.

The treatment is complex and requires short term control of acute symptoms followed by long term stabilization and flare prevention with minimal side effects [2, 13,14].

## Case report

We present the case of D.F., a 21–year–old female patient, with generalized erythematous skin lesions, flexural lichenifications accompanied by intense pruritus, painful fissures and erosions resulting from scratching. The whole body was covered with fine branny scales. She also presented erythematous plaques with thin scales on the scalp. The onset of cutaneous symptoms lasted for 3 years before the admission in our clinic with xerosis and erythematous and edematous plaques on the arms, neck and face, accompanied by intense pruritus. She was ocasionally treated with oral antihistamines but the lesions progressed in time on the trunk and legs. The scratching led to erosions and lichenification followed by painful fissures on the neck , anterior torax and at the flexures.

**Fig 1:**
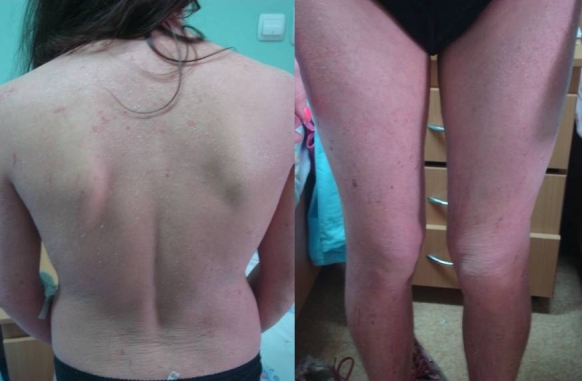
First presentation : eritematous-edematous , veziculous , extensive lesions  with lichenification and erosions from scratching

The patient had no personal or familial history of allergic rhinitis or asthma. She had no other chronic medical conditions and was not on any medication. Physical examination, revealed a strong pulse, with a regular heart rhythm and a rate of 84 bpm, blood pressure of 110/60 mm Hg, weight of 54 kg. There were no signs of lymphadenopathy.(Fig 1)

The complete blood count (CBC) showed leukocytosis (a white blood cell count of 11,3 × 10^3^/µL) with high serum eosinofilia (2,440/µL), and very high total IgE serum (11449UI/L). Skin punch biopsy showed moderate acanthosis and spongiosis and dermal infiltration composed of lymphocytes, hystiocytes and eosinophils. In atopic dermatitis, histopathology is nonspecific, being useful for differential diagnosis.

Differential diagnosis in this case, included other conditions that could lead to exfoliative erythrodermic syndrome: erythrodermic psoriasis, lymphoma, leukemia, cutaneous drug reaction, allergic contact dermatitis, seborrheic dermatitis, pityriazis rubra pilaris and pemphigus foliaceus.

After definite diagnosis, the treatment was started promptly. She received a high dose of systemic corticosteroids–Prednisone 40mg (0,75mg/kgC) with gradual tapering of dose with 5 mg every 3 days, to a daily dose of 20 mg, then with a slower decrease, 5 mg every week, to prevent the rebound of symptoms. She also received a 7 days systemic antibiotherapy and oral antihistamines. Intensified skin hydration and topical corticosteroids were instituted during the tapering period, in order to suppress the rebound flaring associated with the narrow UVB band (311nm).

**Fig 2:**
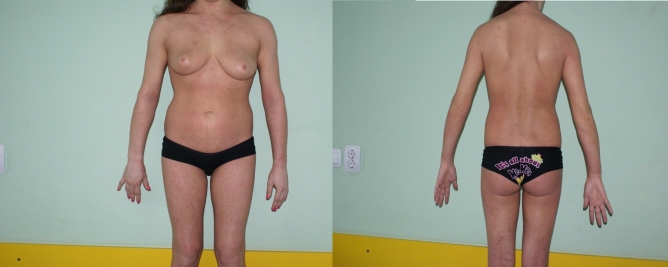
After 2 weeks of treatment : considerable improvement of lesions

The evolution was very good, with a complete clearance of lesions in about six weeks. After the cessation of systemic therapy and topical corticosteroids we insisted on a long term maintenance therapy with adequate cleaning and hydration of the skin, avoiding the irritating factors and potential allergens. (Fig 2)

## Discussions

Exfoliative erythroderma is a serious, possible life threatening reaction. There is considerable heat dissipation and fluid loss due to the dilatation of capillaries. Moreover, high output cardiac failure, electrolyte imbalance and loss of serum protein may appear due to exfoliation. The patient may feel cold and present pruritus, fatigue, weakness, anorexia, weight loss and malaise. There is a high risk of secondary skin infection with S. aureus and even sepsis. Treatment should be instituted promptly, with supportive measures such as fluid and electrolyte replacement, systemic antibiotherapy if signs of infection are still present in the treatment of the underlying disease. Diagnosis is difficult and it is  based on the history of the preexisting disease and pathognomonic signs and symptoms. [2]

In this case, the late onset of the disease and the lack of personal or familial history of atopic diseases made the diagnosis harder to put. Arguments pro atopic dermatitis diagnosis were intense pruritus, lichenifications at flexures and on the neck and the high IgE serum level. 

We put differential diagnosis with other possible causes of erythrodermic reactions. The graduate onset, and the considerable period of time since the beginning of symptoms excluded a ***cutaneous drug reaction ***. Psoriasis generally presents hyperkeratotic plaques of scalp or knees and elbows. The skin punch biopsy showes parakeratotic hyperkeratosis and polymorphonuclear cells in the epidermis forming microabcesses of Munro in the stratum corneum.*** Severe allergic contact dermatitis*** presents with an eruption that starts in a sensitized patient after repeated exposures to the allergen at the site of exposure with ulterior generalization. ***Sezary syndrome*** is a variant of cutaneous T cell lymphoma presenting erythroderma, peripheral lymphadenopathy and cellular infiltrations of atypical lymphocytes in the skin and blood. It usually occurs in patients over 60 years old. ***Pemphigus foliaceus*** may present as exfoliative erythroderma. It is a superficial form of pemphigus, with acantholysis in the granular layer of epidermis due to the circulation of autoantibodies to a 160–kDa intercellular antigen (desmoglein 1), in the desmosomes of keratinocytes. 

The intensive treatment, the lack of other chronic medical conditions and the good cooperation with the patient helped in the complete resolution of lesions without other complications or side effects. Systemic anti–inflammatory treatment for the acute phase is rapidly effective, but long–term use should be avoided, as side effects are inevitable. Topical therapy should be instituted with a tapering of the doses in order to prevent the rebound flaring.

Considering that atopic dermatitis is a chronic relapsing disease, it is very important that the patient understands her condition in order to have an adequate life style and, in the mean time, to avoid unnecessary measures and constrains [3,13].

The skin must be thoroughly cleansed with non-irritant and low allergic formulas. The bath should be short and the use of bath oils is necessary to avoid dehydration. Topical emollients should be applied directly after the bath for a better penetration, after the gentle drying of the skin.  Emollients should be used twice, daily, in order to reestablish and preserve the hydrolipid film of the skin, and restore the epidermal barrier function, which is able to prevent water loss and allergen penetration trough damaged skin [14]. Studies show that moisturizers used regularly decrease the need for topical corticosteroids [3].

Avoiding the irritants such as detergents, chlorine in the pool water, rough clothing fabrics or wool is necessary because they can contribute to the alteration of skin barrier favorising water loss [3]. In addition, the environmental temperature and humidity should be moderate to avoid excessive sweating. It is important to control the exposure to allergens such as house dust mite or animal dander. Individual food allergies should be investigated and incriminated aliments should be removed from the diet [8]. Contact sensitizations should be diagnosed and implicated factors avoided [15,16].

A broad set of immunomodulatory agents have been used for severe atopic dermatitis refractory to other therapies, but their use is limited because of systemic toxicities and high costs. Cyclosporine A is a systemic calcineurin inhibitor used after organ transplantation. Multiple studies demonstrated that patients with refractory disease benefit from short–term cyclosporine treatment. However, the disease relapses rapidly after cessation of therapy. This therapy is associated with systemic side effects such as elevated serum creatinine level, renal impairment or hypertension. Topical calcineurin inhibitors–Tacrolimus and Pimecrolimus–are safe and effective therapy for children of 2 years old and older and for adults. The most frequent side effect was a burning sensation at the application site. Due to the fact that it may restore the immunologic Th1–Th2 imbalance, the recombinant interferon– gamma, showed persistent long term improvement. However, the wide use has been limited by its high cost and difficulty in predicting responders. Intravenous immunoglobulin showed good responses and should be considered in refractory cases, but the major disadvantage is the high cost [3,13].

## Conclusions

It is very important to recognize atopic dermatitis as a cause of erythroderma, especially in a patient with late onset of the disease and no personal or familial history of allergic diseases. Moreover, it is of high interest to treat it promptly and to prevent ulterior recurrences by educating the patient to have an adequate life style and to treat the flares at the very first symptoms.

## References

[1] Hanifin JM, Rajka G (1980). Diagnostic features of atopic dermatitis. Derm Venerol.

[2] Freedberg IM, Eisen AZ Fitzpatrick's Dermatology in General Medicine.

[3] Bouguniewicz M, Eichenfield LF (2003). Current management of atopic dermatitis and interruption of atopic march. J. Allergy Clin Immunol.

[4] Isolauri E, Turjanmaa K (1996). Combined skin prick and patch testing enhances identification of food allergy in infants with atopic dermatitis. J. Allergy Clin Immunol.

[5] Darsow U, Ring J (2000). Airborne and dietary allergens in atopic eczema: a comprehensive review of diagnostic tests. Clin Exp Dermatol.

[6] Niggermann B (2001). The role of atopy patch test in diagnosis of food allergy in infants and children with atopic dermatitis. Pediatr Allergy Immunol.

[7] Roehr CC, Riebel S (2001). Atopy patch tests, together with determination of specific IgE levels, reduce the need for oral food challenge in children with atopic dermatitis. J Allergy Clin Immunol.

[8] Bindslev–Jensen C (2001). Standardization of double blind, placebo-controlled food challenges. Allergy.

[9] Darsow U, Laifaoui J (2004). The prevalence of positive reactions in the atopy patch test with aeroallergens and food allergens in subjects with atopic eczema: a European multicenter study. Allergy.

[10] Ring J, Kunz B, Bieber T (1989). The ‘atopy patch test’ with aeroallergens in atopic eczema. J Allergy Clin Immunol.

[11] Darsow U, Vieluf D, Ring J (1995). Atopy patch test with different vehicles and allergen concentration. An approach 
to standardization. J Allergy Clin Immunol.

[12] Hoare C, Li Wan Po A (2000). Systematic review of treatments for atopic eczema. Health Technol Assess.

[13] Darsow U, Lubbe J, Taieb A, Seidenari S, Wollenberg A, Calza AM, Giusti F, Ring J (2005). Position paper on diagnosis and treatement of atopic dermatitis. JEADV.

[14] Loden M, Andersson AC (1999). Improvement in skin barrier function in patients with atopic dermatitis after treatment with a moisturizing cream (Canoderm). Br J Dermatol.

[15] Manzini BM, Ferdani G (1998). Contact sensitization in children. Contact Dermatitis.

[16] Mortz CG, Andersen KE (1999). Allergic contact dermatitis in children and adolescents. Contact Dermatitis.

